# Ten simple rules for failing successfully in academia

**DOI:** 10.1371/journal.pcbi.1010538

**Published:** 2022-12-15

**Authors:** Stefan Gaillard, Tara van Viegen, Michele Veldsman, Melanie I. Stefan, Veronika Cheplygina

**Affiliations:** 1 Center of Trial and Error, Utrecht, the Netherlands; 2 Center for Education, University Medical Center Utrecht, Utrecht, the Netherlands; 3 Independent Scientist, Amsterdam, the Netherlands; 4 Wellcome Centre for Integrative Neuroimaging, Department of Experimental Psychology, University of Oxford, Oxford, United Kingdom; 5 Centre for Discovery Brain Sciences, Edinburgh Medical School: Biomedical Sciences, The University of Edinburgh, Edinburgh, United Kingdom; 6 ZJU-UoE Institute, Zhejiang University, Haining, China; 7 Department of Human Medicine, Medical School Berlin, Germany; 8 IT University of Copenhagen, Copenhagen, Denmark

## Abstract

Failure is an integral part of life and by extension academia. At the same time, failure is often ignored, with potentially negative consequences both for the science and the scientists involved. This article provides several strategies for learning from and dealing with failure instead of ignoring it. Hopefully, our recommendations are widely applicable, while still taking into account individual differences between academics. These simple rules allow academics to further develop their own strategies for failing successfully in academia.

## Introduction

Failure is part of our lives and professional careers. Academic life is no exception. Rejected papers, experiments gone wrong, or letting somebody down, have probably happened to all of us. The scientific process itself often relies on formulating hypotheses and testing them, and often seeing that our original ideas do not hold up. Yet in some ways, some academics can often still pretend that failure does not exist. This can lead to negative feelings in other academics, possibly with life-changing consequences such as mental health problems.

Initiatives to talk about failure come up from time to time, with the CV of Failures [1] being one of the most common ones. While they have good intentions, they may themselves miss the target. Often, an iceberg image is used to illustrate the CV of Failures (see, for instance, [2]). The top of the iceberg represents the successes, when in fact most of the iceberg is hidden and consists of failure, hard work, and privilege, among others. But what about all the other ice we don’t see? In fact, 98% of the world’s ice is in glaciers, not in icebergs. Of the remainder, lots of smaller pieces of ice are not “real” icebergs and are instead called “bergy bits” or “growlers.” So perhaps the CV of Failures is not an accurate representation of reality either: We are missing the people who never even got a chance.

Instead, we take a more general approach of talking about failure, discussing what it means to us, strategies that have been useful, and advice we wish we would heard years ago. We do not want to suggest everybody needs to follow the same approach—we simply want to share perspectives that might be useful to other (early career) academics.

## Rule 1: Define failure

What do we mean by failures? The original “CV of Failures” mostly equates failures to rejections [3], but that seems too simplistic a definition. There are various dimensions that might be worth looking at, when deciding when something is a failure or not, for example:

- Failure is setting a goal and subsequently never reaching the goal. This differentiates failures you might only get 1 chance at (such as doing your PhD at a specific place) from errors that you can still correct, such as errors in your experiments.
- Failure can be useful or not useful [4]. In useful failures, called brilliant by innovation researcher Paul Iske, the result is different than intended, but still valuable—for the goal in question, or for another purpose. Failures that do not contribute to reaching a goal are not considered useful in this framework. An example of such a failure is accidentally knocking over your glassware in the laboratory, thereby ruining your whole experiment. Everybody knows they are not supposed to knock over their glassware, and such a mistake seemingly provides little to learn from. However, others might argue that even this instance of failure provides information, such as that you are tired or should organize your workbench differently.
- Failure as something you have a degree of influence on (or not). For example, even with an excellent CV and project idea, you can still fail to secure a grant, simply because by definition not everybody can succeed. Moreover, we do not have control over others’ opinions (in this example, grant reviewers or applicant letter writers, which influences the outcome), but this is part of life and career.
- Failure as an event versus failure as a state. Say a paper from a big project in your lab is still unpublished after a year of rejections. Some may consider this state of being unpublished as a failure. Others may instead look at each individual rejection from journals as failure events. This latter view is more actionable. For instance, you can review what went wrong with a particular submission and map out changes to make for the next one.

As the authors of this paper, we initially tried to create a taxonomy of failures, but we soon realized that we identified the same events as different types of failures or disagreed on whether they were failures at all. What qualifies as a failure for one person might not qualify as a failure for someone else. Moreover, what you identify as a failure or a mistake might change over time (for example, depending on career stage and can therefore change with the evolution in our perspective on failure). To fail successfully, we recommend that readers start by identifying what failure means to them, which failures they have encountered, and how these failures affected them. It may be useful to think not only of one’s definition of failure, but also of one’s definition of success at the same time, since the 2 are often related. Setting and reaching goals could be one such definition.

We also would like to emphasize that these definitions might vary in scale and extend beyond our academic work, and include things like “feeling guilty for spending too much time at the lab,” and “being proud of prioritizing your health.” In that sense, “shadow CV” might be a better term for what is commonly referred to as “CV of Failures.”

## Rule 2: Dare to fail

To fail successfully, you first need to start somewhere and dare to try things that might end up failing. As an example, it is better to test a hypothesis in an experiment and failing, than not doing the experiment at all. Do not be discouraged by the deceiving look of a traditional CV. A traditional CV looks straightforward, but chances are most colleagues did not have a linear trajectory to building their career. Even though it might look like a planned path straight to success while building their career, in reality, it is often a bumpy road. Moreover, one should remember that each career stage can take anywhere between 3 to 8 years. As such, early career researchers might overestimate the impact of failure on the career in its entirety, because the short-term impact is larger than the impact on the eventual career. We encourage early career researchers to find out which opportunities are there for you, and apply—although failure is common, you might also be surprised by the result.

Both successes and failures are often linked to serendipity, which might sound like magic that “just” happens to you, but [5] writes it is important to notice these opportunities. Sometimes accepting an opportunity that might not be the most logical for your career, or rejecting an opportunity that is, might lead to unexpected events, with more interesting turns of your career as a result.

Another way to get some experience with failing is intentionally doing something you are not good at yet, such as playing a musical instrument or bouldering. You don’t have to have the goal to “succeed” here either in the sense of winning a prize for your performance—aim for getting better yourself, even if in absolute terms you are always “losing.”

## Rule 3: Don’t compare yourself to others

Playing an instrument or bouldering brings up an important point of running your own race. Comparing yourself to your peers is a dangerous game—there is a growing body of literature linking social comparison to unhappiness [6] and impostor syndrome [7]. Comparisons are fundamentally flawed. For one, because they rely on unequal information: The information you have about your own professional endeavors is more complete than the information you have about those of your peers. As we mentioned in the introduction, even the failures people present to the world are carefully curated and do not tell the whole story. People sharing their failures often do so with hindsight and from a position of ultimate success and privilege, which may not be the position that you yourself are currently in.

Instead of comparing yourself to others, focus on your own journey. Useful practices include goal-setting and reflection [8]. Since success in academia is often dependent on factors outside of your control, it is useful to focus on process, not outcomes, for instance, by setting implementation intentions [9]. For example, you could focus on writing every day or submitting 2 grants per year, and you would be successful if you achieve this goal, independent of the outcome of the paper or grant. These implementation intentions can span different timescales.

## Rule 4: Do compare yourself to others

While it is important to run your own race, sometimes it does help to also look at what your peers are doing. This is especially useful if your peers are open about their own setbacks and failures. It is important to acknowledge that both being allowed to fail and being in a position to talk about it is a privilege which not everyone is afforded [10]. But those who can talk about their failures have increasingly done so over the last decade or so: Scientists publish their CVs of failure [1,3,11] or contribute to initiatives such as the “How I Fail” blog [12] (where academics discuss different types of failures and successes that extend beyond the CV) and the #FailureFriday hashtag (https://twitter.com/hashtag/failurefriday). In the startup world, there is a parallel tradition of “Fuckup Nights” [13] aimed at encouraging openness towards failure. Seeing that others experience failure can help to put your own setbacks into perspective [14], and it may help to read or talk about other people’s coping strategies.

Do think more broadly about who your peers might be. Your current academic bubble does not include everyone who did a PhD in your field. Peers that chose different career paths might inspire you, as there might be positions outside of academia that did not exist a few years ago. Even within your own lab, there might be scholars that have a career trajectory that is not comparable to yours. Find peers that resonate with the path that you would like to pursue. There are many publications with relevant advice and resources, such as [15–17] or the blogs From PhD To Life [18] or Beyond The Professoriate [19].

## Rule 5: Keep track of failures and successes

You need to have a good idea of everything you have done in the past, whether successful or not. You might think it is sufficient to keep track of only the big milestones on your CV, as that is the only thing that “counts,” and try to ignore the failures as much as possible. However, there are 3 problems with this approach.

Not having an overview of what you’ve spent time on might give you a biased view of your efforts/value of your work. Due to the numbers game big successes will be rare, so focusing only on those could make it seem like you are not putting in enough work. Keeping track of what you’ve spent time on, even if it doesn’t result in a big line on your CV, can be helpful to appreciate that.

Keeping track of more “everyday” things that can go wrong, like a bug in your code or an experiment gone wrong, is also useful for many reasons. In some disciplines, this is commonly done through lab notebooks, but the concept of logging what you are working on each day and what the outcomes are (rather like a captain’s log book) can also help to appreciate your efforts. Having such a practice could lead to useful insights on what to do next and could make these topics more approachable to discuss with your peers.

It is important to also keep track and celebrate small things that are positive, but that might not be visible on a traditional CV, such as thank you email from a student. For this, some people have suggested the creation of a “happy folder” in your e-mail inbox [20] that you can come back to and look at every so often. Note-taking apps where you can add different types of notes (photos, PDF documents, forwarded emails) are also suitable for this purpose.

Another reason to have an overview of everything is to be able to have a meeting with yourself where you evaluate a period of time and adjust your plans for the future. You might notice, for example, that your time was too disjointed between different commitments, and you were not able to complete several of them—without either a success or failure decision in the end. There are many systematic ways to track your work, such as the Getting Things Done [21] system, where you use the same trusted tools at regular time intervals. If you track what you work on each week, the big successes and failures will be easier to add to this overview than vice versa.

## Rule 6: Study the system

Through tracking your own successes and failures, and (sometimes) comparing them to others, you might get a more complete picture of the academic system you are in. It might be a good idea to learn more about it, as you will get a better idea of how the system has benefited or disadvantaged you and others, putting all successes and failures in perspective.

One such factor is the overreliance of academic research on publishing significant results and how this distorts incentives. When exploring new ideas, we would expect some of them to be wrong—something to build upon, as [5] writes. However, not confirming an initial hypothesis is often seen as a “negative result,” not worth publishing. This is seen as negative by universities, grant agencies, and others who decide a researcher’s career. On the other hand, when a field is more developed, it is easier to estimate which ideas will yield results, and thus get publications, grants, and so forth. Many researchers stay in this plateau, and as a result, progress in the field slows down [22].

We also need to understand failure in relation to privilege and discrimination. There is a vast amount of evidence documenting how underrepresented groups have reduced odds of paper acceptance, funding, or promotion—see [23,24] for some specific examples. Game theoretical models and empirical studies show that minorities are likely to get less credit for articles [25–27], even when such groups have been found to contribute more to innovation within science [28]. Even though the system is stacked against them, members of underrepresented groups will still experience failure events as individual failures. On the other hand, members of privileged groups not only have lower odds of failure, but also they are afforded more opportunities to bounce back from failure and try again. If we conceptualize failure as just something that happens at an individual level, we miss such system-wide dynamics and biases. Promoting inclusive metrics of success and impact is one of the first steps in tackling these system-wide dynamics and biases [29].

It is also worth noting that the system we are in determines how we perceive success and therefore, failure. Within academia, there are few career paths, and narrow definitions of success, for instance, related to publication, citations, and grant income. But success can mean many more things, both within academia [30] and, even more so, outside of it [31]. At universities, ways of redefining success include moving away from metrics as measures of success and appreciating a wider range of academic activities [32,33].

Better understanding the system can facilitate structural change. The occurrence of failure provides a signal that something needs to change, either relating to the goals or behavior of the individual, or relating to the environment. Environmental change requires brave individuals willing to be the first to enact change—to build new systems that challenge existing assumptions and value and reward a diverse range of merits.

## Rule 7: Make failure a part of the process

Learning from failure often happens after a mistake has occurred, but it can also be part of the process. For example, engineers constantly learn from failure, since prototypes are in a way failed versions of the final product. In science, peer review has traditionally had the role of being the error-correcting mechanism, although there are various flaws. For example, increases in journal submissions combined with overtaxed reviewers lead to reviewers not spending enough time on reviews—making it more likely for them to overlook flaws and mistakes [34].

However, there are more tools that we have at our disposal. In some fields, it is now not unusual to post a preprint on arXiV or a similar service before submitting to a journal, see for example [35], and possibly asking for feedback in the body of the preprint. This gives you a chance to incorporate other ideas or references (or even, having other researchers join as co-authors if they made significant contributions) before the start of the formal review process [36].

Another example is red teams, a concept best known for their use in fields like (cyber)security, the military, and intelligence agencies. Red teams simulate enemy attacks by finding and exploiting flaws in their employer’s systems, so that these vulnerabilities can be resolved before actual negative consequences occur. Recently, academic red teams [37] have started to emerge, with the goal of helping researchers spot mistakes in their research before their articles go through peer review. The advantage is that it often takes less resources to prevent failure than correct it—for example, revising the methodology before the experiments are conducted. This method turns research into a more streamlined process of trial and error: By identifying mistakes along the way, failure is a fundamental part of the process. This is in line with the idea of a growth mindset [38] and believing that your skills in learning from failure are not innate, but can be developed over time.

You might be surprised at what happens when making failure a part of the process, and letting others know that you have done so, even if it is just sharing frustrations about spending hours on fixing a bug in your code with another researcher. Vulnerability shows courage and can help you connect with others and develop a sense of belonging [39].

## Rule 8: Create a support network

Failure happens to everybody, and this is something you can use to your advantage. Talking about your situation with others can be helpful at any stage of the process: before doing something that might potentially fail to weigh your options and get advice, during to adjust the course if necessary, or afterwards, either to celebrate or cry it out, and to learn from the experience.

However, although everybody fails, it might be difficult to find a group of people where you feel comfortable sharing your thoughts. For example, as a PhD researcher, you might fear your supervisors would disapprove of your plans about a career outside academia (which is not a failure [31], but may be perceived as such by others). Fortunately, you don’t need to rely on formal mentorship structures and can find support in many other places [40], which might even give you ideas on how to approach the subject with your supervisor when you are ready. More advice on building inclusive mentor–mentee relationships can be found in [29,41].

A great way to connect with others in similar situations is through online channels, and this is even more true during the pandemic. You could think of joining online communities with a Slack group (or similar), for example, NewPISlack (https://twitter.com/newpi_slack) for early career principal investigators. Such groups typically have channels where you can ask for advice on different topics. Another option is to connect with people directly through Twitter, for example, by following people who often use hashtags like #PhdChat or #AcademicTwitter. Please note that you can use social media to find people, but that you can take your interactions elsewhere, such as Slack, Zoom, etc. For a tutorial, if you are unsure about using Twitter, or would like to weigh the pros and cons of social media, please see [42,43].

We compiled more resources where one might find people or ideas to connect with in Table 1.

**Table 1:** Examples of resources that might help you learn more about failure and/or find other people who have experienced similar struggles.

Category	Title/URL	Description
Podcasts	Human Risk	Scientists, politicians, policy makers, etc. talking about the role of risk in human life, often related to failure
	Changing Academic Life	People sharing their (real) stories, failure is not always central but the interviews often touch on (downsides of) academic culture
	The Other F Word	General interviews on tough things in life (not only academics), some pressures will be recognizable for all
	Story Collider	Stories from academics, both about science and life challenges
	Voices of Academia	Academics sharing their stories related to mental health and well-being
	How to Fail with Elizabeth Day	Inspirational stories about overcoming failure, can be a motivation boost
Blog posts	Growing Up in Science	“Unofficial” stories of scientists
	How I Fail	Interviews with scientists about different aspects of failure
Communities	Graduate Student Slack	Support for everything related to being a graduate student
	Future PI Slack	Informal peer mentoring group, predominantly for biomedical postdocs aiming to become principal investigators (PIs)
	New PI Slack	Community of new PIs, predominantly in North America
	Open Life Science	A training and mentoring community in open science, related to life sciences
	The Turing Way Community	A community dedicated to collaborative, reusable, and transparent research
Other	Red Team Market	Hire a team to detect potential errors in your scientific work
	Instituut voor Brilliante Mislukkingen	Information, workshops, and more to understand different types of failures (original in Dutch)
	Journal of Trial and Error	Outlet to publish “failed” research

## Rule 9: Find what works for you

Not everybody will be comfortable with the same approach of dealing with failure, and as the authors, we disagreed what types of strategies worked for us. Therefore, we would also advice the reader to research other strategies, and adopt the ones that might resonate more, depending on the circumstances.

One strategy that might be worth considering is gamifying failure. You might want to collect “points” for each rejection you receive, and aim for a certain number of rejections per year [44]. This can train you to take each individual rejection less seriously and have a celebration to look forward to if you do reach your rejection goal. There is, of course, a trade-off here between quality and quantity, and you will need to decide what is the minimum quality you are willing to accept, while still keeping track of the total time you spend applying. In the How I Fail series, Melanie Stefan suggests a slightly different strategy: buy a lottery ticket every time you submit an application.

Some failures like deleting all your data might not be insightful, so you might proactively try to avoid them. Here, lessons can be learned from fields where failure has high costs. In “The Checklist Manifesto,” Atul Gawande [45] describes how using checklists has reduced the number of deaths in aviation and in surgery. By having multiple people follow steps systematically, such as checking that equipment is working correctly, there is less room for accidents. Similar ideas could be applied to the research and publication process, to accepting projects and finishing them on time, and so forth. You can create such checklists for yourself (even if you think you will remember how to do something the next time), or, even better, share them with team members.

**Fig 1:**
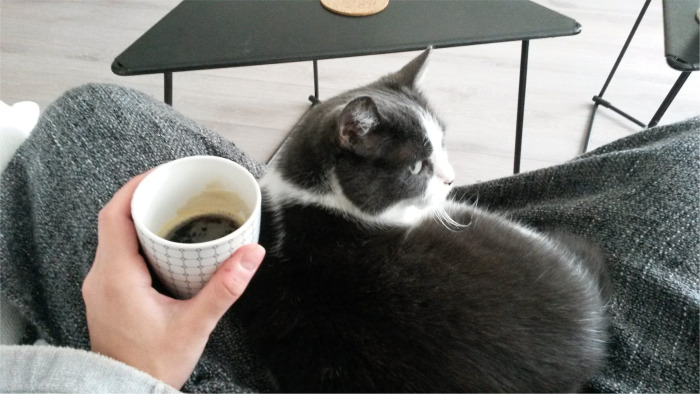
Example of giving yourself time to process failure.

Whether insightful or not, many failures are also unavoidable and difficult to frame in a positive light. In this case, consider treating yourself as a good friend, Kristin Neff advises in “Self compassion” [46]. Remember that if you failed or were not good enough at something, is not the same as being a failure or not good enough in general. Do not push yourself to try again immediately, and give yourself time to process the event first. Spending time with your cat (Fig 1) or an “emergency beer” with a friend can help as well.

## Rule 10: Pay it forward

Now you’ve had some failures and learned from them. What can you do next? Pay it forward, of course. If you are up for it, you could share your CV of Failures, or participate in an interview series like How I Fail [12] or Growing Up In Science [47], or Twitter hashtags like #FailureFriday. Understandably, sharing publicly on the internet may not be for you. You can also implement sharing failures in other ways, for example, by dedicating part of a lab meeting to it, or using the resources in Table 1 to engage with other academics by offering your support when they fail and normalizing rejection by sharing your personal failures in more private channels.

You can also be a mentor to somebody who has not learned all of this about failure yet. You can do this without any formal mentorship structure, and even if you are not in a senior position—you can be mentors with a peer as well (see Rule 8 for ideas on finding such people). This is both a great way to pay it forward, and for you to re-frame your own experiences.

Lastly, find ways in which you can go against practices that perpetuate a biased, exclusionary system. The fact that everybody else keeps on doing it is not enough—if everybody hides behind the incentives, things are not going to change [48]. Perhaps you are not in a position to fully make a 180 degree turn, but even little things like publishing a preprint, in expectation will help to normalize such practices. Of course, “Ten Simple Rules” papers on how to change the system would be highly welcome as well.

## Conclusion

Although failure means different things to individual academics, we have aimed to give general advice on how to deal with failure throughout your career—always taking into account that each person operates in different circumstances. Generally, we recommend that academics build a network that facilitates open discussion about failure. Embracing failure as part of the scientific process can even help to prevent future failures or detect errors in your current work. While building and engaging with this network, it is important to keep in mind the extend to which failure should be attributed to yourself and how the system influences when failure occurs and even what is considered failure. Above all, individual academics should set their own goals and work towards those, not letting themselves be held back by expectations of or comparisons to others. We hope our 10 simple rules will allow academics to fail successfully in their future careers.
